# Proposal for field sampling of plants and processing in the lab for environmental metabolic fingerprinting

**DOI:** 10.1186/1746-4811-6-6

**Published:** 2010-01-29

**Authors:** Tanja S Maier, Jürgen Kuhn, Caroline Müller

**Affiliations:** 1 Department of Chemical Ecology, Bielefeld University, Universitätsstr. 25, 33615 Bielefeld, Germany

## Abstract

**Background:**

Samples for plant metabolic fingerprinting are prepared generally by metabolism quenching, grinding of plant material and extraction of metabolites in solvents. Further concentration and derivatisation steps follow in dependence of the sample nature and the available analytical platform. For plant material sampled in the field, several methods are not applicable, such as, e.g., collection in liquid nitrogen. Therefore, a protocol was established for sample pre-treatment, grinding, extraction and storage, which can be used for analysis of field-collected plant material, which is further processed in the laboratory. Ribwort plantain (*Plantago lanceolata *L., Plantaginaceae) was used as model plant. The quality criteria for method suitability were high reproducibility, extraction efficiency and handling comfort of each subsequent processing step.

**Results:**

Highest reproducibility of results was achieved by sampling fresh plant material in a solvent mixture of methanol:dichloromethane (2:1), crushing the tissue with a hand-held disperser and storing the material until further processing. In the laboratory the material was extracted threefold at different pH. The gained extracts were separated with water (2:1:1 methanol:dichloromethane:water) and the aqueous phases used for analysis by LC-MS, because the polar metabolites were in focus. Chromatograms were compared by calculating a value Ξ for similarities. Advantages and disadvantages of different sample pre-treatment methods, use of solvents and solvent mixtures, influence of pH, extraction frequency and duration, and storing temperature are discussed with regard to the quality criteria.

**Conclusions:**

The proposed extraction protocol leads to highly reproducible metabolic fingerprints and allows optimal handling of field-collected plant material and further processing in the laboratory, which is demonstrated for an exemplary field data-set. Calculation of Ξ values is a useful tool to judge similarities between chromatograms.

## Background

The sum of all metabolites and their specific concentrations are representative for the physiological state of an organism in a particular environment under defined conditions. Metabolomics techniques, and especially metabolic fingerprinting [1], can provide an insight into the variability between biological samples exposed to different environmental conditions [2,3]. The main problem of measuring the metabolome lies in the vast range of very small to very large chemical compounds differing highly in physico-chemical properties, e.g., molecular weight, partial molar volume, polarity, boiling- and melting point, functional groups, reactivity and three-dimensional structure [4]. Furthermore, the concentration of individual metabolites ranges from a few molecules to molar concentrations in a single cell or organism. Metabolomics requires repeatable, reproducible and robust analyses of all metabolites across a broad dynamic range [2,5-7]. Up to now no extraction technique or protocol and analytical platform is able to fulfil all of these requirements. Thus, for practical reasons only a part of the metabolome is analysed in metabolomics experiments.

The number of protocols for initial quenching of the metabolism, extraction and analysis of different target metabolites, metabolite profiles or metabolic fingerprints of various organisms is numerous and still growing [4,6,8,9]. In most cases the metabolism is quenched by shock-freezing of plant tissue in liquid nitrogen, followed by the grinding of the frozen material and the extraction with different solvent mixtures [10-12]. Alternatively shock-freezing might be followed by lyophilisation (see, for example, [13-15]). Extraction with cold methanol is well established to gain polar and medium polar metabolites, e.g., iridoid glycosides of Plantaginaceae [16,17]. Also often mixtures of methanol, chloroform and water are used to extract metabolites from various organisms, resulting in a biphasic separation [10,18,19]. The aqueous phase is thereby freed of lipids and phospholipids and can be analysed separately [20,21].

Most metabolomics studies are carried out in the laboratory under highly controlled conditions and with all instruments at hand. However, protocols for sampling of biological tissue in the field to investigate the chemical variation characteristic for plants grown under natural environmental conditions are lacking. In the field, samples usually cannot be placed immediately in liquid nitrogen for quenching of the metabolism. Legal regulations for transportation of liquid nitrogen are hard to fulfil for most labs, and liquid nitrogen evaporates even from relatively large tanks in a shorter time period than a field sampling excursion may take. Similar limitations apply for dry ice. However, at ambient temperature, faced under field-sampling conditions, reactions of metabolites including enzymatic transformations may still occur and alter the metabolic fingerprint. To circumvent this problem field samples may be stored in a solvent with low reactivity in a dark place [12]. The direct extraction of fresh plant material in solvents with quenching capabilities is a more applicable alternative in the field. Another option is the drying of plant material at ambient temperature (see, for example, [16,22-24]).

Further processing steps involve destruction of cell structures and homogenisation of the sample. Cells often disintegrate and the sample can easily be ground by ball mills by freezing the material in liquid nitrogen [12,25]. The grinding of unfrozen fresh plant tissue may result in changed metabolic fingerprints due to ongoing metabolism, and the exposure of ground material to air causes unwanted reactions. To avoid these reactions, the tissue can be homogenised in organic solvents. Depending on the analytical platform used for metabolite analysis, extraction methods with high selectivity and specificity for a certain group of metabolites are desirable. In analysis of polar metabolites by liquid chromatography comparably non-polar solvents may distort the analysis. In contrast, separation by gas chromatography of polar and non-volatile compounds is not feasible and may harm the chromatographic columns.

In this paper a protocol is presented that is applicable for plant tissue extraction of field-collected samples for metabolic fingerprinting with LC-MS in the laboratory. All relevant processing steps of plant material sampling and pre-treatment, grinding, extraction (solvents, pH, extraction frequency and duration, phase separation, temperature), sample storage and data processing were subsequently established, optimising one step after the other. The criteria for optimisation of the sampling protocol were mainly reproducibility, extraction efficiency and handling comfort (practicability). It was focused on medium polar to polar metabolites of the plant metabolome. Ribwort Plantain, *Plantago lanceolata *L. (Plantaginaceae), was used for the experiments as it is a widespread plant [26]. Optimisation of the field sampling protocol was realised with plants grown under defined conditions in the lab to test for reproducibility of extraction conditions. Different methods of pre-treatment of samples, grinding, solvent use and phase separation are discussed. Moreover, a parameter (Ξ) is introduced, which provides a measure of similarity between two chromatograms of independently processed samples. This value can be used to test the reproducibility of the applied extraction method. The final protocol was used to test its usage for analyses of metabolic fingerprints of field-collected material from different localities.

## Methods

### Plant cultivation and general sampling procedure

*Plantago lanceolata *plants were grown in a climate chamber at 22°C, 16:8 h light-dark-cycle and 70% relative humidity. Seeds (Rühlemann's Kräuter und Duftpflanzen, Horstedt, Germany) were germinated in small pots (diameter 5 cm) with potting soil (Fruhstorfer Erde, Archut, Germany). Two weeks after germination plants were transferred individually into flowerpots (diameter 13 cm). Plants were watered regularly and were grown without additional fertilisation.

Every step of the following basic extraction procedure was tested in an individual experiment to investigate effects of a given parameter (see also Table 1, Table 2 and Table 3). Optimised parameter choice was then used for establishment of the subsequent experiment. The general experimental procedure is outlined in the following and changes to this general procedure are described in the subsections for each experiment. Several equal-sized middle-aged leaves of one *P. lanceolata *plant (six to eight weeks old, not flowering) were pooled for every experiment, if not described otherwise. The plant material was chopped with scissors into small pieces of about 5 × 5 mm within 10-15 s to gain a homogeneous leaf batch. The batch was then distributed in samples of 100 ± 5 mg. For first extraction, each sample was ground in 2 ml solvent in 15 ml Falcon tubes with a hand-held electrical disperser (Polytron PT 1600 E, Co. KINEMATICA AG, Luzern, Switzerland) at a speed of 15,000 min^-1 ^until the plant particles had a homogeneous size (between 30 and 90 s). Two further extraction steps followed with 2 ml each of the respective solvent mixture. After every extraction step the samples were vortexed for 30 s and centrifuged for 10 min at 3,863 rcf (Rotanta/S, Co. Hettich, Bäch, Switzerland). The supernatants were gathered and pooled. Every alternative processing step was replicated five times (unless mentioned otherwise).

**Table 1:** Differences in PCA scores between differently pre-treated samples.

Pre-treatment	Scores of PC 1 (mean ± sd)		Scores of PC 2 (mean ± sd)	
Fresh leaves	-1.68 ± 1.06	A	2.21 ± 1.01	a
N_2_	-3.24 ± 4.41	B	0.94 ± 1.00	ab
Lyophilisation	-0.23 ± 1.89	AB	-0.69 ± 1.53	ab
Air-dried	-2.21 ± 0.54	AB	-4.09 ± 4.75	b

**Figure 1:**
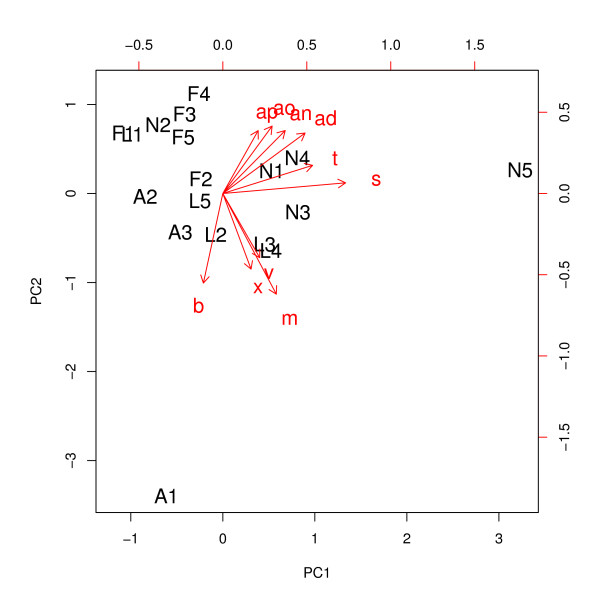
**Effects of pre-treatment on metabolite pattern**. Influence of sample pre-treatment on metabolic fingerprints of *Plantago lanceolata*. Biplot of loadings and scores of principal component analysis of peaks achieved with HPLC of leaf samples extracted in 100% methanol. For clarity some peaks are omitted. F = processing of fresh plant material, N = material frozen in liquid nitrogen, L = material frozen and lyophilised and A = air-dried samples. Number of replicates N = 5; air-dried samples N = 3. Freshly processed leaves show low scattering on first and second principal axis (PC 1 and PC 2), which explain most of the variances (31 and 26%, respectively) within the data set. Low scattering indicates high reproducibility. For statistical comparisons see Table 1.

Loadings (mean ± standard deviation) of PC 1 and PC 2 of a PCA on metabolic fingerprints of the different pre-treatment methods (see Figure 1). *Plantago lanceolata *leaf material was either extracted directly, frozen in liquid nitrogen, lyophilised or air-dried for 24 hours at room temperature, before further extraction in 100% methanol. Extracts were analysed by HPLC. Number of replicates N = 5; air-dried samples N = 3. Differences among pre-treatments were tested with ANOVA followed by Tukey's HSD tests. Different upper case letters indicate significantly different scores of PC 1 (*F*_3,14 _= 3.121, *P *< 0.05); lower case letters indicate significantly different scores of PC 2 (*F*_3,14 _= 4.238, *P *< 0.01).

**Table 2:** Extraction efficiency of repeated extractions.

	Peaks [%] at t_*R *_= 0 - 14 min (mean ± sd)	Peaks [%] at t_*R *_= 14 - 30 min (mean ± sd)	Peaks [%] at t_*R *_= 30 - 38 min (mean ± sd)	Peaks [%] at t_*R *_= 0 - 38 min (mean ± sd)
1. extraction	3.02 ± 0.59	77.64 ± 7.69	19.34 ± 8.17	41.61 ± 3.76
2. extraction	1.65 ± 0.46	53.47 ± 3.17	44.88 ± 3.20	64.91 ± 3.06
3. extraction	0 ± 0	34.68 ± 10.79	65.32 ± 10.79	72.04 ± 3.21
4. extraction	0.06 ± 2.86	28.82 ± 5.44	71.13 ± 5.50	87.75 ± 1.09
5. extraction	3.56 ± 2.48	31.50 ± 9.73	66.82 ± 12.38	94.93 ± 0.59
6. extraction	2.05 ± 1.13	30.68 ± 6.49	67.39 ± 4.86	98.07 ± 0.17
7. extraction	1.79 ± 0.68	29.54 ± 4.98	68.66 ± 3.59	100 ± 0

Percentage (mean ± standard deviation) of metabolites (peaks) detected in 7 subsequent extractions of *Plantago lanceolata *leaf material, extracted in 2:1 methanol:dichloromethane and analysed by HPLC. Number of replicates N = 5. Columns 1-3 show the percentages of peaks eluting between different retention times (t_*R*_) within one extraction step. Columns 1-3 add up horizontally to 100% for each extraction step. In the fourth column the sum of all 150 integrated peaks of all 7 extractions was set as 100%. This column indicates the cumulative values from one extraction to another.

**Table 3:** Effects of solvents, extraction duration, phase separation and storage on extraction reproducibility and efficiency.

	Duration for first extraction	Ξ (mean ± sd)		Number of metabolites (mean ± sd)	
**a) Solvents**					
1:0 CH_3_OH:CH_2_Cl_2_	0 h	0.74 ± 0.02	a	1544 ± 49	A
	1 day	0.85 ± 0.03	b	1980 ± 47	B
	1 week	0.71 ± 0.02	c	574 ± 23	C
3:1 CH_3_OH:CH_2_Cl_2_	0 h	0.78 ± 0.03	d	1677 ± 46	D
	1 day	0.77 ± 0.02	d	647 ± 16	E
	1 week	0.75 ± 0.02	ade	602 ± 23	E
2:1 CH_3_OH:CH_2_Cl_2_	0 h	0.83 ± 0.02	d	1730 ± 52	F
	1 day	0.86 ± 0.05	bef	616 ± 13	CE
	1 week	0.72 ± 0.02	c	605 ± 12	CE
1:1 CH_3_OH:CH_2_Cl_2_	0 h	0.82 ± 0.03	g	1712 ± 52	DF
	1 day	0.88 ± 0.01	f	2040 ± 20	G
	1 week	0.72 ± 0.01	ac	649 ± 25	E
					
**b) pH**^*a*^					
pH 2 - pH 2 - pH 2		0.81 ± 0.06	abc	1501 ± 20	AB
pH 2 - pH 6 - pH 9		0.85 ± 0.16	cd	1509 ± 16	AB
pH 2 - pH 9 - pH 6		0.90 ± 0.03	e	1513 ± 28	AB
pH 6 - pH 6 - pH 6		0.91 ± 0.04	e	1538 ± 29	AB
pH 6 - pH 2 - pH 9		0.93 ± 0.02	e	1545 ± 14	A
pH 6 - pH 9 - pH 2		0.89 ± 0.02	de	1380 ± 226	B
pH 9 - pH 9 - pH 9		0.81 ± 0.26	bc	1498 ± 10	AB
pH 9 - pH 2 - pH 6		0.80 ± 0.05	bc	1510 ± 29	AB
pH 9 - pH 6 - pH 2		0.79 ± 0.02	b	1505 ± 46	AB
					
**c) Phase separation method of three extraction steps**					
A) Extraction in CH_3_OH:CH_2_Cl_2_, H_2_O added to pooled supernatant (2:1:1)		0.99 ± 0	a	1662 ± 14	A
B) Extraction in CH_3_OH:CHCl_3_, H_2_O added to pooled supernatant (2:1:1)		0.93 ± 0.02	b	1594 ± 20	B
C) Extraction in CH_3_OH:CH_2_Cl_2_, H_2_O added to every supernatant (2:1:1)^*b*^		0.97 ± 0.01	a	1627 ± 18	AB
D) Extraction in CH_3_OH:CH_2_Cl_2 _(2:1), removal of aqueous phase and addition of new solvent mixture after every extraction step^*b*^		0.97 ± 0	a	1656 ± 23	A
					
**d) Storage**^*c*^					
-80°C		0.85 ± 0.04	a	831 ± 5	A
4°C		0.91 ± 0.01	b	849 ± 25	A

^*a *^Subsequent extraction steps with the solvent mixture methanol:dichloromethane 2:1 of different-pH.

Addition of 0.1% formic acid and of 0.1% ammonia solution respectively was used to adjust pH to 2 or 9.

The untreated solvent mixture had a pH between 6 and 6.5.

^*b *^Two parts of H_2_O were added again to the final pooled supernatant.

^*c *^Samples were kept after first extraction in 2:1 methanol:dichloromethane at room temperature for 1 week until further processing. Completely processed extracts were stored at different temperatures before analysis.

Similarities between chromatograms, calculated as Ξ values (pairwise comparisons, formula (2)) and number of metabolites (peaks) (mean ± standard deviation) in chromatograms of replicate extractions (N = 5, for Table 3b: N = 3) of *Plantago lanceolata *leaf material. Leaf samples were extracted threefold whereat the first extraction step was kept at room temperature for one week before further processing (if not noted otherwise, see a). Samples were analysed by LC-MS. The closer Ξ is to 1, the more similar are the resulting chromatograms. Differences between Ξ values and number of metabolites were calculated by ANOVA followed by Tukey's HSD tests. Different lower case letters indicate significantly different Ξ values (a): *F*_3,16 _= 3.24, b) *F*_8,19 _= 2.48, c) *F*_3,16 _= 3.24, d) *F*_1,8 _= 5.32, for all *P *< 0.05), different upper case letters refer to significantly different numbers of metabolites (a): *F*_3,16 _= 11.38, b) *F*_8,19 _= 0.96, c) *F*_3,16 _= 5.36, d) *F*_1,8 _= 1.77, for all *P *< 0.05).

In general, the solvent mixture for extraction was 2:1 methanol:dichloromethane (both LC-MS grade, Co. Fisher Scientific UK Limited, Loughborough, Great Britain), unless otherwise noted. After three extractions in this mixture, one part water was added to the pooled supernatants to initiate phase separation (total ratio: methanol:dichloromethane:water 2:1:1). After shaking and centrifugation, the aqueous phase was analysed by HPLC-TOF-MS (1200 Series HPLC, 6210 Time-of-Flight, Agilent Technologies, Santa Clara, USA) (see below). Due to logistic reasons several samples could be measured with HPLC-DAD only. All samples were stored until analysis at -80°C. The remaining pellets of the samples taken from the field in Bielefeld, Augustdorf and Hövelhof were dried for one week under a fume hood and weighed to determine the approximate dry mass of the extracted leaf material.

### Pre-treatment of sampled plant material

To investigate the influence of leaf treatment prior to extraction, plant samples were collected and pre-treated in different manners. Aliquots of a homogeneous *P. lanceolata *leaf sample were either used as freshly cut material, frozen in liquid nitrogen directly after cutting, frozen in liquid nitrogen and lyophilised overnight (Leybold-Heraeus LYOVAC GT 2, Co. SRK Systemtechnik GmbH, Riedstadt/Goddelau, Germany) before further processing, or fresh leaves put in paper bags and air-dried for 24 h at room temperature. The fresh material was homogenised immediately with a hand-held disperser in methanol in a Falcon tube. Shock frozen, lyophilised and air-dried samples were pulverised in 2 ml Eppendorf tubes with a ball mill (MM 301, Co. Retsch GmbH, Haan, Germany) with 3 balls (diameter 4 mm) at a speed of 32 s^-1 ^for 30 s before extraction in 100% methanol. Extraction was done threefold in methanol. Neither water nor dichloromethane were added to these samples. The extracts were analysed by HPLC-DAD.

### Grinding

The effects of grinding methods on quenching and extraction efficiency were tested by grinding either with a ball mill or a hand-held disperser. Fresh leaf samples were ground in a ball mill with 3 balls in 2 ml Eppendorf tubes for 30 s at a speed of 32 s^-1^. Grinding beakers were pre-cooled at -18°C. Solvent was added afterwards. Alternatively fresh leaves were filled together with 2 ml solvent (2:1 methanol:dichloromethane) in a Falcon Tube and ground with a hand-held disperser at a speed of 15,000 min^-1^until the plant particles had a homogeneous size. Homogenised plant material was air-dried to measure particle size after grinding.

### Extraction

In order to test the number of times the material needs to be extracted to gain almost quantitative extraction of metabolites, leaf pieces were extracted seven times instead of only three times, according to the general experimental procedure (see above) in methanol:dichloromethane (2:1). After every extraction step the respective supernatant was analysed without further phase separation by HPLC-DAD to determine the amount of metabolites extracted from the plant material in each step separately. For every chromatogram, peaks were automatically integrated by standard settings of the ChemStation software (see 'Data analyses'). The sum of all 150 integrated peaks over all seven extractions was set to 100%.

Percentage of the sum of peak integrations for every extraction step was calculated.

In a subsequent experiment, different mixtures (1:0, 3:1, 2:1 and 1:1) of methanol and dichloromethane as extraction solvents were tested. Additionally, the effects of time samples kept after the first extraction before further extraction were tested for each of the four different solvent mixtures. From one large batch of plant material, five samples for each solvent mixture and time-point were ground once with a hand-held disperser and material remained therein for 0 h, 1 day or 1 week until further extraction at room temperature. After the second and third extraction step, the aqueous supernatants of all three steps were pooled. Water was added for a final phase separation and samples were analysed by LC-MS.

Furthermore the influence of storing temperature on the metabolic fingerprint of *P. lanceolata *extracts was tested. Fresh plant samples ground in methanol:dichloromethane (2:1) (first extraction) were either left at room temperature for one and for two weeks, respectively, or stored for one or two weeks in a cooling chamber at 4°C before further processing (subsequent two extractions followed). To exclude influences of phase separation no water was added to these samples before analysis by LC-MS.

Threefold extraction in methanol:dichloromethane (2:1) at three pH (2,6,9)in different order was used to estimate the influence of pH on reproducibility and extraction efficiency. Formic acid (98-100%, Co. Merck, Darmstadt, Germany) was added to acidify the solvent mixture (pH 2) and ammonia solution (Carl Roth GmbH & Co., Karlsruhe, Germany) was added to basify it (pH 9). The untreated solvent mixture had a pH between 6 and 6.5. Threefold extraction of each sample was performed with either three times solvent of identical pH or with either one of all six possible combinations containing acidic, neutral and basic solvent in different orders (N = 3 per combination) (see Table 3). Samples were stored in the first extraction solvent for one week at 4°C before phase separation and further processing (two subsequent extractions) followed according to the general extraction procedure. The samples were analysed by LC-MS.

### Phase separation

Efficient phase separation and influence on metabolic fingerprints was tested with four techniques. A) Samples were extracted in methanol:dichloromethane (2:1), supernatants were pooled and water was added (2:1:1 methanol:dichloromethane:water) to the pooled supernatants. B) The same was done with 2:1 methanol:chloroform as extraction solvent. C) After every extraction step with 2:1 methanol:dichloromethane water was added (2:1:1 methanol:dichloromethane:water), both phases were removed and pooled separately. Two parts of water were added again to the aqueous phase for a final phase separation. D) After the first extraction with 2:1 methanol:dichloromethane water was added (2:1:1 methanol:dichloromethane:water), the aqueous phase was removed and new solvent mixture (methanol:dichloromethane (2:1)) was added to the remaining organic phase. Again the upper phase was removed and pooled with the first extraction step. This was repeated one more time. Two parts of water were added again to the pooled supernatant at the end for a final phase separation. The aqueous phases of all four treatments (A-D) were analysed by LC-MS.

### Storage of samples

Effects on plant metabolic fingerprints of final extract storage in a freezer at -80°C versus storage in a cooling chamber at 4°C before analysis were tested. Samples were extracted threefold with 2:1 methanol:dichloromethane. After the first extraction, samples were stored at room temperature for 1 week until further processing. Phase separation took place as described in method A in subsection 'phase separation'. Fully processed extracts were kept cold or frozen for one week and were then analysed by LC-MS.

### Field application of the most suitable method

To test the practical application of the most suitable method, leaf samples of *P. lanceolata *were collected from field-sites within one day in May 2008. The oldest and youngest leaf of non-flowering plants (between 5 and 11 cm height) in a distance of at least 10 cm and up to 25 m were harvested at fields with different land-use in Augustdorf (51°54' 06" N, 08°43' 16" O at centre) and Bielefeld (52°00' 12" N, 08°32' 25" O at centre), Germany. In Augustdorf one field was used as a horse pasture (N = 17 samples collected), while another one was unused and mowed once per year (N = 15). In Bielefeld one field was also an unused meadow mowed once per year (N = 21), whereas the other meadow was used as a recreation area (N = 20). The differentially treated fields (used/unused) at the two sites were in close vicinity to each other (in Augustdorf about 120 m, in Bielefeld about 300 m) to ensure comparable environmental conditions apart from land-use within the sites. The average temperature during harvest (Augustdorf 28.3°C, Bielefeld 25°C) and the soil differed between sites (Augustdorf: sandy, Bielefeld: argillaceous).

The oldest and youngest leaf of each plant were pooled to average leaf age effects. Plant material was cut with scissors and brought into 4 ml solvent (2:1 methanol:dichloromethane, pH 6) within a few seconds. Samples remained in the initial solvent for one week at 4°C, then a second extraction followed with the same solvent but at pH 2 and a third extraction at pH 9. Supernatants were pooled and phase separation initiated by addition of water (2:1:1 methanol:dichloromethane:water). Fresh weight was not determined in the field, because no appropriate balance could be brought to the field and it was necessary to insert leaf material in solvent as fast as possible. Instead, the extracted pellet was dried for one week in the lab. Dry weight (between 27.3 and 252.5 mg) of every sample was used for normalisation after data analysis. Samples were analysed as described below.

### Instrumentation/Chromatographic conditions

For the analysis by HPLC and LC-MS a Grom-Sil 120 ODS-4-HE-column (150 × 2 mm, 3 *μ*m; Alltech Grom GmbH, Rottenburg-Hailfingen, Germany) was used. A gradient from water with 0.1% formic acid (98-100%, Co. Merck, Darmstadt, Germany; solvent A) to acetonitrile (LC-MS grade, Co. Fisher Scientific UK Limited, Loughborough, Great Britain; solvent B) with 0.1% formic acid with a flow of 0.3 ml was used, starting at 5% B with a hold for 2 min and going from 2-34 min to 95% B with a hold for 2 min at 95% B, followed by a cleaning and column equilibration cycle. Column oven temperature was set to 35°C. Measurement was in positive mode with a Dual ESI source (drying gas flow: 11 l/min, gas temperature: 350°C, nebuliser pressure: 45 psi). Reference masses 121.050873 (purine, [C_5_H_4_N_4_+H]^+^) and 922.009798 (HP-0921, [C_18_H_18_O_6_N_3_P_3_F_24_+H]^+^) in positive mode were used for internal mass calibration during the runs, introduced by a second sprayer in the source (API-TOF reference mass solution kit, Agilent Technologies, Santa Clara, USA). The fragmentor voltage was at 175 V, capillary voltage was at 3,500 V, the skimmer at 65 V. DAD signals were integrated at 254 nm. Prior to and after an analysis sequence a blank and a reference sample of *P. lanceolata *were measured to assure instrumental reproducibility. The analytical replicates (reference samples) were prepared from a pooled bulk of *P. lanceolata *leaf material sampled near Bielefeld University (Germany) in 2007 and extracted 3 fold in 100% methanol. The methanol extract was stored at -80°C. ¿From analytical replicate measurements five peaks occurring in all samples with intensities between 1·10^3 ^and 1.25·10^6 ^counts and a distribution over the chromatographic range were randomly chosen for calculation of relative standard deviation (RSD) to estimate machine variance. A needle-wash step was included between sample injections to reduce carry-over.

### Data analysis

Data were exported from the respective software of the HPLC (ChemStation, Version Rev. B.02.01 [244]) and the LC-MS (MassHunter, Version B.01.03) and analysed by the free software environment for statistical computing and graphics, R (version 2.7.0 and newer) [27]. For the HPLC data the auto-integration function of the ChemStation software was used (calculates a value for peak-width based on the run time and optimum detection criteria; provides a 10:1 signal-to-noise-ratio). LC-MS data were pre-processed with the "xcms" package of R [28], which nonlinearly aligns retention time and accurate mass of LC-MS produced peaks [29], in a time range of 0-38 min. The parameters method = "centWave", ppm = 23, profmethod = "bin", peakwidth = c(20,75), snthresh = 10, prefilter = c(3,200), fitgauss = T were used for peak finding with "xcmsSet". For the grouping of the found peaks (command "group") the used settings were bw = 30, minfrac = 0.5, minsamp = 1, mzwid = 1, max = 50, sleep = 0.

Peak areas were analysed per 100 mg fresh weight. Data were transformed by logarithmic calculus. Pre-processing functionality of the MeltDB platform [30] was used in order to compute sets of common and distinctive peaks in our replicate measurements of the different extraction conditions.

The formulas (1) and (2) were applied for peaks generated by "xcms" as a measure for similarity of peaks and chromatograms:(1)

*ξ *(equation (1)) is a measure for the similarity of a single compound's peak in two chromatograms. *P*_*i, min *_is the integration (area) of the smaller peak i (i = 1 to n) of one chromatogram and *P*_*i, max *_the integration of the larger peak i (i = 1 to n) of the other chromatogram. With this equation the amounts of a metabolite in two sample chromatograms can be compared. For all pair-wise comparisons of chromatograms of samples measured with LC-MS the peaks with values lying between the 0.25- and 0.75-quantile were used for scaling the respective chromatograms to the higher values. Scaling was necessary to diminish effects of instrument variation.

Ξ (equation (2)) is a measure for the similarity of two chromatograms. All single peak comparisons are summed up and divided by the number of peaks to provide an average peak similarity, which equals the similarity of the respective chromatograms. Each peak (including possible fragments and adducts of single peaks) was assumed to correspond to one metabolite for simplicity. Ξ = 1 indicates 100% similarity of chromatograms and thus high reproducibility of the extraction method, Ξ = 0 indicates no similarity. For further statistical analysis with Principal Component Analysis (PCA) and ANOVA the R package "stats" [27] was used to compare similarities between treatments. Resulting Ξ-values and numbers of metabolites (peaks) of the chromatograms were compared by ANOVA, followed by Tukey's HSD test, because data were normally distributed (tested by Kolmogorov-Smirnov test) and variances were homogeneous (tested by Levene test). For calculation of PCA z-scores were used. Differences between PCA scores of metabolic fingerprints were tested by ANOVA followed by Tukey's HSD test (pre-treatment of samples) and by Kruskal-Wallis-tests, followed by *post-hoc *paired comparisons tests, when data were not normally distributed (field-sampling data from four field sites).

## Results

Analytical replicates showed variability between measurements of several weeks. Peaks from measurements of the analytical replicates at retention times of 66 s (182.96233 m/z), 174 s (163.03805 m/z), 245 s (359.14848 m/z), 1273 s (353,26811 m/z) and 1518 s (326.37742 m/z) had 6.1%, 4.6%, 12.2%, 8.5% and 7.2%% RSD, respectively. The mean Ξ value calculated for 21 analytical replicate measurements measured over several weeks was 0.97 ± 0.05. For the replicate samples measured within one day, the mean Ξ value of the chromatograms was 0.98 ± 0.04.

### Pre-treatment of sampled plant material

PCA-scores of freshly processed plant samples showed low variation along the first and second principle axis (PC 1 and PC 2), which explained most of the differences (57%) between metabolic fingerprints due to different pre-treatment (Figure 1). Harvested leaf material frozen in liquid nitrogen clustered comparably close on PC 2 but scattered on PC 1. Scores of lyophilised samples spread on PC 1 and PC 2 over a wider range (about -0.8 to 0.8) and air-dried samples dispersed widest on PC 2 (-3.4 to 0). Wide scattering in the PCA biplot indicates low reproducibility. Significant differences were found in PC 1-scores between the extracts of fresh leaves and shock frozen material in liquid nitrogen (ANOVA followed by Tukey's HSD test, *F*_3,14 _= 3.121, *P *= 0.020; Table 1). PC 2-scores were significantly different between the extracts of fresh leaves and air-dried material (ANOVA followed by Tukey's HSD test, *F*_3,14 _= 4.238, *P *= 0.004; Table 1).

### Grinding

Grinding of fresh leaf pieces in a ball mill resulted in squeezed plant material. No satisfactory homogenisation could be achieved. In contrast, a homogeneous crushing of leaf material was obtained using the hand-held disperser. Leaves were shredded with this equipment to a maximum particle size of 1 mm (dried material).

### Extraction

With regard to efficiency of repeated extraction steps, less than half of all metabolites could be extracted in the first extraction step (Table 2). Efficiency increased with number of extractions. With a threefold extraction more than 70% of metabolites were extracted (Table 2). Not until the fifth step more than 90% of the metabolites could be extracted. In general more non-polar metabolites (eluting later, retention time 30-38 min) could be gained with later extraction steps.

**Figure 2:**
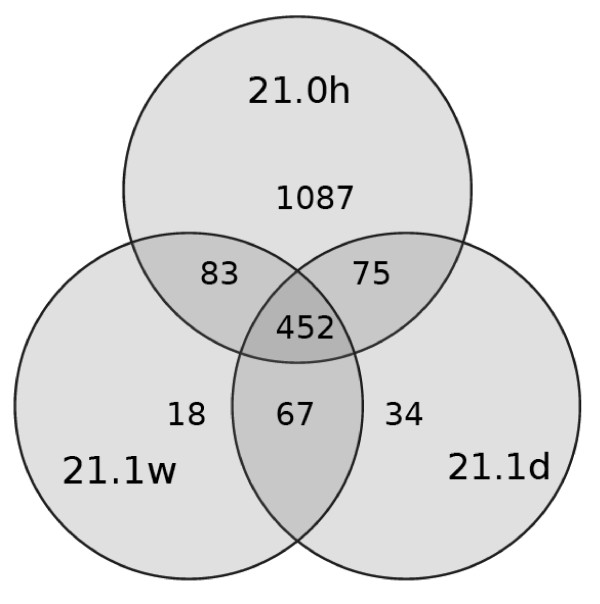
**Time effects on extractable metabolites**. Venn Diagram of the results of the extraction with a mixture of 2:1 methanol:dichloromethane and three extraction durations of 0 h (21.0 h), 1 day (21.1 d) and 1 week (21.1 w), respectively. In brackets the total number of metabolites of the single samples is given. 452 metabolites were found in every sample. For details of extraction see legend of Table 3.

**Figure 3:**
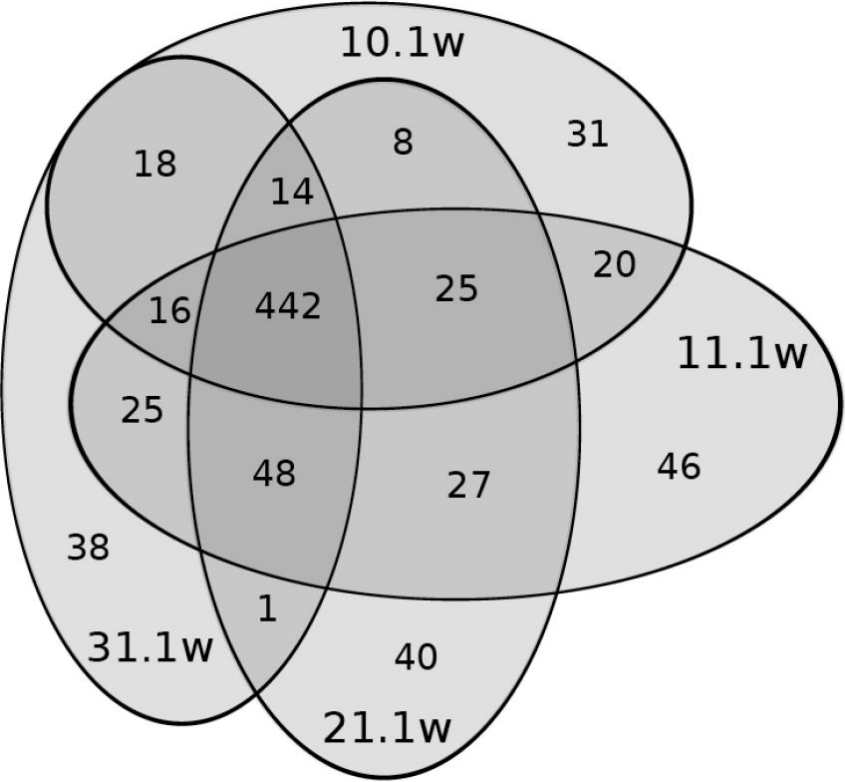
**Solvent effects on metabolite extraction efficiency**. Venn diagram of the results of extractions with either 100% methanol (10.1 w, 580 metabolites), 3:1 (31.1 w, 602 metabolites) or 2:1 (21.1 w, 605 metabolites) or 1:1 (11.1 w, 649 metabolites) methanol:dichloromethane. Leaf material of *Plantago lanceolata *was ground in the respective solvent mixtures once and remained therein for 1 week until further extraction at room temperature. Phase separation by addition of water was initiated after the second and third extraction step and aqueous supernatants pooled. 442 metabolites were found in every sample. For details of extraction see legend of Table 3.

Extractions in methanol:dichloromethane at different ratios resulted in a different number of detectable metabolites as well as in different reproducibility within one extraction method, calculated as Ξ values. After the immediate threefold extraction (0 h), most metabolites were extracted in a 2:1 mixture, followed by a 1:1 (18 metabolites less) and 3:1 mixture (53 metabolites less) (Table 3). Fewer metabolites were found in extracts processed further after one day. When extracts were processed further after one week, less than half of the metabolite number was detectable compared to analyses of immediately extracted samples (Figure 2). About 85% of the peaks from samples measured after one day or one week could be found as well in the samples analysed immediately after extraction. Approximately 5% of the found peaks occurred only in the samples extracted for one day and one week. In samples further processed after one week, most metabolites were extracted in a 1:1 mixture, the least number was extracted in 100% methanol. The lowest standard deviation of peak numbers was evident in the 2:1 mixture of samples processed at this time point. Three fourth of the overall extracted metabolites could be gained with every solvent ratio (Figure 3). About 7% of the extracted metabolites were unique for the respective extraction solvent ratios. The Ξ values revealed different reproducibility of different extraction methods. Significantly highest similarity between samples could be gained with a 1:1 solvent mixture and samples kept after first extraction for one day. Reproducibility was in general higher for extractions in a 2:1 and 1:1 mixture than for extractions in a 1:0 and 3:1 mixture. When Ξ values were calculated for different sample-groups (different solvents and/or different extraction durations among each other), mean Ξ values were between 0.17 and 0.35.

Keeping samples between first extraction step and further extraction and processing either at room temperature or at 4°C had no significant influence on the number of metabolites or the similarity of metabolic fingerprints (data not shown).

Extractions in a 2:1 solvent mixture of methanol:dichloromethane at different pH revealed different results with regard to reproducibility of sample extraction and metabolite numbers (Table 3). The treatments that started with the first extraction at pH = 9 showed on average the lowest Ξ values. An intermediate Ξ and therefore reproducibility was found when samples were first extracted with solvent at pH = 2. The solvent order neutral-acid-basic revealed the significantly highest Ξ values. This extraction procedure also resulted in the highest number of metabolites. Generally lower numbers of metabolites were found when the pH of the solvent was the same in every one of the three extraction steps.

### Shaking and phase separation

All shaking methods (Table 3) resulted in a suitable phase separation. The two phases were clearly separated and the aqueous phase was large enough to be removed, except for the organic phase in the 3:1 mixture, which was fairly small. The aqueous phase in all extraction treatments was nearly or completely colourless.

Phase separation and pooling of the aqueous phase after every extraction (technique C) and the addition of new solvent mixture to the organic phase and phase separation after every extraction (technique D) were time consuming, because many more processing steps were necessary. Shaking of the pooled aqueous phases of all three extraction steps was more convenient.

The mean Ξ value (Table 3) and thus similarity between chromatograms was highest for phase separation technique A, where phase separation was initiated for the pooled supernatants at the end of the processing. No significant differences were found between Ξ values of the three methods where dichloromethane (A, C and D) was used. The significantly lowest Ξ was obtained for a solvent mixture of 2:1 methanol:chloroform (B). The average highest number of metabolites was found with technique A and lowest with B.

### Field application of the most suitable method

**Figure 4:**
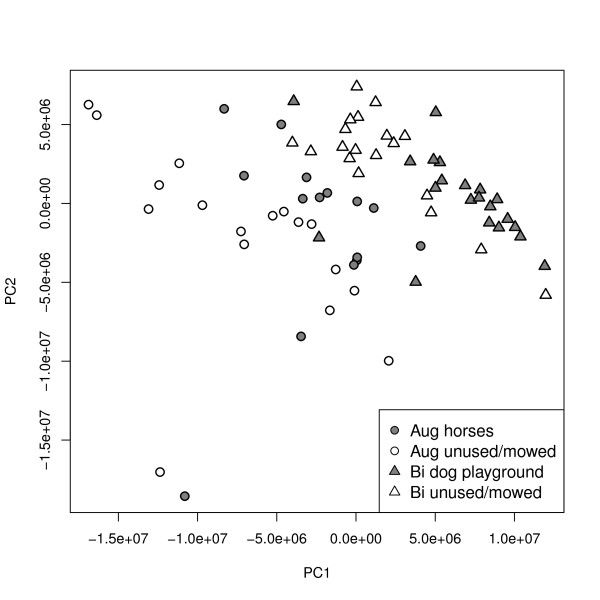
**Results of field sampling at different sites with different land-use**. Scores plot of principal component analysis of chromatographic peaks of *Plantago lanceolata *field-sampled at four plots, two in Augustdorf (Aug) and two in Bielefeld (Bi), each with different land-use. Leaf samples were extracted following the recommended protocol (Table 4) and analysed by LC-MS. N = 73 plant samples were taken. At PC 1, explaining 86% of the variance within the data, samples from Augustdorf with horses and Augustdorf unused/mowed were significantly different and samples from Bielefeld were different to samples from Augustdorf with horses (Kruskal-Wallis test followed by *post hoc *paired comparisons tests, *P *< 0.01). All sites and treatments were significantly different at PC 2, explaining 11% of the variance, (Kruskal-Wallis test followed by *post hoc *paired comparisons tests, *χ*^2 ^= 488.349, *df *= 71, *P *< 0.001).

Metabolic fingerprints of plants sampled from Augustdorf and Bielefeld showed significant differences (Kruskal-Wallis test, *χ*^2 ^= 488.349, *df *= 71, *P *< 0.001) in a PCA at PC 1, explaining 86% of the variance within the data set (Figure 4). Furthermore, samples from the unused field in Augustdorf were significantly different from the unused field in Bielefeld at PC 1 (*post hoc *paired comparisons test, *P *< 0.01). Within Augustdorf samples from the unused meadow and the horse pasture could be distinguished significantly (PC 1, *P *< 0.01). The effects of land use was not significantly different within the two sites, but values of samples from both sites and treatments were significantly different at PC 2 (*P *< 0.001), which explained 11% of the variance within the data.

## Discussion

For sample preparation of field collected material for an environmental metabolic fingerprinting approach a robust method was developed. The criteria for the choice of the most suitable extraction procedure of field-collected material were first of all reproducibility (the conservation of abundance/number of peaks from the same sample), and furthermore efficiency (number and abundance of peaks, which were assumed to be compounds or metabolites) and handling comfort in the field. Reproducibility of extraction method and resulting chromatograms is a necessary prerequisite for comparison of plant samples grown under different conditions or at different sites, which is displayed in changes of metabolic fingerprints. Sample collection and storing conditions were adapted to the typical situation of field trips, where liquid nitrogen and a freezer may not be available and it may take another week before a laboratory is reached for further adequate sample processing.

The pre-treatment of plant material had a high influence on the metabolic fingerprint. In the lab shock-freezing of leaves in liquid nitrogen is the preferred method for metabolic fingerprinting, because the metabolism can effectively be quenched, the frozen material can easily be ground and further extraction and processing is possible [10-12]. However, for field-trips most scientists might encounter difficulties to comply with legal requirements for transportation of liquid nitrogen for longer distances and the nitrogen will quickly evaporate. Storage on dry ice might be an alternative. However, samples freeze only slowly on dry ice compared to shock-freezing in liquid nitrogen, and dry ice can also only be used for short field-trips but will usually not last for one week. Collection of fresh material directly in solvent or air-drying of plant material are therefore potentially suitable alternatives for field sampling as a compromise. Samples of freshly processed leaves clumped most in a PCA biplot (Figure 1), indicating highest reproducibility. Extracts of freshly processed leaf material had low variance - and thus high similarity - of scores in the first and second principal components (PC 1 and PC 2). All other methods showed high variances on at least one principal component's scores (Figure 1, Table 1). Therefore, collection of fresh material directly in solvent is the preferred method of pre-treatment for samples collected in the field, where neither nitrogen nor dry ice is available. Lyophilisation of samples is a method often used for studies of target metabolites [31], as sample dry mass can be exactly determined and leaf material can be stored easily for later grinding and extraction. However, drying of samples may change the metabolite pattern to a large extent especially due to irreversible adsorption of metabolites on cell walls and membranes [25]. Also, a lyophiliser cannot easily be brought to the field sampling site. When extracting fresh material, masses of sampled material can be approximately determined by weighing the dried pellet after extraction, as was done in this study.

Quenching of the metabolism can be reached by cutting the leaf material with a hand-held, electric disperser directly in the solvent mixture. The saw-teeth homogenise the leaf material to very small pieces and destroy the cell walls mechanically. But a preceding manual cutting of the leaves in pieces of about 5× 5 mm with scissors is important, because the dispenser cannot process whole leaves of *P. lanceolata*. The cutting process takes only a few seconds but still changes of metabolites with high turnover rates and hence of metabolic fingerprints could occur [12]. The necessary manual pre-cutting of leaf material definitely is a drawback of this approach. The impact of time needed for cutting on metabolic fingerprints remains to be tested. From a practical point of view, the disperser can be plugged to an electrical generator or an electrical inverter converting DC electricity from sources like (car) batteries, solar panels or fuel cells to AC electricity at the sampling location. Grinding material with ball mills is not a reasonable option in the field, as these devices are big and difficult to carry along. Moreover grinding of fresh leaf pieces in a ball mill resulted in squeezed plant material cleaved to the bottom and the top of the Eppendorf tubes and thus insufficient homogenisation and quenching.

In general, the extraction procedure should be quantitative for any metabolite in the final sample mixture. In many metabolic fingerprinting or metabolite profiling studies, only one extraction is carried out (see, for example, [14,32,33]). However, one extraction resulted in less than 50% of metabolites (peak integration) in *P. lanceolata *samples, which is not sufficient. About 90% of metabolites could only be extracted after the fifth extraction (Table 2). Three extraction steps, which resulted in extraction of about three fourth of the total metabolite number, seem a useful compromise between handling time (which is rather high for five or more extractions) and extraction efficiency. Furthermore, the highest number of metabolites was gained from *P. lanceolata*, when all three extraction steps were done immediately in a row (Table 3). However, when plant material is sampled outdoors, it is usually impossible to accomplish several extraction steps in a row. Therefore, the effects of time between first and subsequent extractions, i.e., how long the plant material was kept in the initial solvent mixture, were tested at 4°C. Storing samples for one week in the first solvent is likely the most suitable method from a practical point of view, despite loss of a high peak number (Figure 2). Possibly the most reactive metabolites have undergone transformations resulting in a relatively inert extract after one week and thus more reproducible analysis results with the drawback of "loosing" some metabolites that may be of major importance.

The choice of the solvent for extraction is a crucial step in metabolite profiling and metabolic fingerprinting studies and might highly depend on the biological material and the metabolites of interest. Often, cold methanol is used for the extraction of polar compounds [12], but also various solvent mixtures were tested and evaluated for extraction qualities in metabolomics studies (see, for example, [11,19,34]). Initial extraction mixtures of methanol and dichloromethane or methanol and chloroform provide high metabolism quenching capability [12]. This also allows later phase separation by addition of a small amount of water to partition the majority of non-polar metabolites such as lipids.

The shaking with water is essential for the extraction process of *P. lanceolata *leaves as could be shown by higher Ξ values (demonstrating higher similarity between replicate sample extractions), than samples processed without phase separation (Table 3). Ξ values of samples with phase separation were comparable to analytical replicate measurements indicating very high reproducibility of the method. With respect to peak numbers no significant differences could be found between mixtures containing different parts of dichloromethane (Figure 3, Table 3). Furthermore, extractions in mixtures of methanol with dichloromethane resulted in more reproducible results in comparison to those with chloroform in *P. lanceolata*. For analysis of polar compounds phase separation in methanol:dichloromethane is especially advantageous since the polar metabolites are then in the upper phase, which is accessible without contamination of the lower phase with a pipette while transferring this phase for further processing. After centrifugation precipitates will rest in the organic phase together with non-polar compounds. In general, mixtures of a higher proportion of methanol than chlorinated organic solvent result in better phase separation ratios (more aqueous phase compared to organic phase). With respect to both sample handling and reproducibility of results, the mixture of methanol:dichloromethane 2:1 and addition of 1 part water for phase separation is overall the most suitable extraction process for samples that have to stay in the initial solvent for one week, as usually necessary when field-sampling.

The temperature during storage of first extraction had no influence on the chemical pattern of the samples, at least when comparing storage at cool temperatures (4°C) and room temperature. However, cooling might be necessary if temperatures increase above 22°C. In any case, samples should be stored at a dark place to avoid degradation of light-sensitive metabolites [12].

The first extraction mixture, in which the sample is stored for one week, should preferably be a neutral solvent to prevent possible matrix or metabolite degradation, that can occur at acid or basic pH [18,35]. Subsequent extraction steps may have a different pH to protonate or deprotonate metabolites, which are not well soluble at neutral pH in the aqueous phase, to enhance extraction efficiency. The differences in reproducibility, when extracting in different order of pH, were generally of minor values (Table 3). High Ξ values were obtained for initial extraction with pH 6. With regard to number of extractable metabolites, most could be gained in the extraction order neutral-acid-basic.

Often samples need to be stored after the complete extraction before they can be analysed in the available analytical platform. Storage at 4°C reduces physical changes within the samples (e.g., adsorption, aggregation) to a minimum, but at these temperature conditions chemical reactions may occur [34,36]. In contrast, at -80°C chemical reactions can be avoided, but physical changes can take place more readily. The comparative analysis of samples stored after the final extraction at 4°C or -80°C showed that storage at 4°C led to a higher reproducibility. This is thus the preferred option for metabolic fingerprinting studies with *P. lanceolata*, but long-term effects of storage at these temperatures (for more than one week) need to be elucidated. In both cases the number of metabolites was significantly reduced in comparison to immediate processing of samples.

This protocol was optimised for extraction of *P. lanceolata *leaves and the described amounts and ratios of leaf material and solvents. Smaller or larger sample amounts might result in poor precision for several processing steps, and different ratios of sample amount to extraction solvent might influence extraction efficiency. For other biological material conditions might differ, depending on the given metabolite composition and their physico-chemical properties. Compromises with regard to metabolite number must be taken into account to gain highest reproducibility for analysis of field-collected samples. The protocol was established on standardised material grown in the laboratory. First experiments with field-collected material showed that the method is indeed highly applicable. In spite of all necessary compromises the method is sensitive enough to discriminate metabolic fingerprints of plant samples from different sites having different environmental conditions (e.g., soil, temperature etc.) from samples of the same site but with different treatments (land-use) (Figure 4). Samples from different sites could be discriminated in a PCA at PC 1, whereas different treatments significantly differed at PC 2. This clearly demonstrates the applicability of the proposed protocol for field sampling.

## Conclusions

A robust method is proposed, which is highly reproducible and guarantees efficient extraction of metabolites for a metabolic fingerprinting approach with *P. lanceolata *leaf material. Multiple extractions of ground material with a hand-held disperser with a mixture of 2:1 methanol:dichloromethane at different pH (neutral - acid - basic) followed by phase separation by addition of water fulfilled criteria of precision, efficiency and handling most sufficiently (Table 4). The described method is suitable for sampling of plant tissue in the field when common techniques used in the laboratory are not at hand outside but subsequent processing is possible in the laboratory.

**Table 4:** Method overview.

Step in processing	Most suitable result with:
Pre-treatment	processing of fresh plant material
Grinding	handheld disperser, speed of 15,000 min^-1^
Extraction: solvent mixture	2:1 methanol:dichloromethane
Extraction: efficiency	threefold extraction:
	1. extraction step at untreated pH 6,
	2. extraction step pH 2,
	3. extraction step pH 9
Extraction: influence of time	first extraction step for one week
Extraction: influence of temperature	ambient temperature
Shaking and phase separation	shaking after the extracts of all three extraction
	steps were pooled
Storage of samples	storage of completely processed extracts until analysis at 4°C

Representation of most suitable method (highest reproducibility and handling comfort) for every processing step for metabolic fingerprinting of plant material (*Plantago lanceolata*) sampled in the field.

## Competing interests

The authors declare that they have no competing interests.

## Authors' contributions

TM carried out all extraction experiments and conducted measurements. JK and CM conceived the study and devised the experimental design. LC-MS methods were developed by TM and JK. All authors participated in data extraction and statistical analysis. All authors wrote, read and approved the manuscript.
